# Identification of Age-associated Proteins and Functional Alterations in Human Retinal Pigment Epithelium

**DOI:** 10.1016/j.gpb.2022.06.001

**Published:** 2022-06-23

**Authors:** Xiuxiu Jin, Jingyang Liu, Weiping Wang, Jiangfeng Li, Guangming Liu, Ruiqi Qiu, Mingzhu Yang, Meng Liu, Lin Yang, Xiaofeng Du, Bo Lei

**Affiliations:** 1 Henan Eye Institute, Henan Eye Hospital, People’s Hospital of Zhengzhou University, Henan Provincial People’s Hospital, Zhengzhou 450003, China; 2 Branch of National Clinical Research Center for Ocular Disease, Henan Provincial People’s Hospital, Zhengzhou 450003, China; 3 School of Medicine, Henan Provincial People’s Hospital, Henan University, Zhengzhou 450003, China; 4 Academy of Medical Sciences, Zhengzhou University, Zhengzhou 450001, China; 5 School of Basic Medical Sciences, Zhengzhou University, Zhengzhou 450001, China

**Keywords:** Human retinal pigment epithelium, Proteomics, Aging, Retina, Apoptosis

## Abstract

Retinal pigment epithelium (RPE) has essential functions, such as nourishing and supporting the neural **retina**, and is of vital importance in the pathogenesis of age-related retinal degeneration. However, the exact molecular changes of RPE during **aging** remain poorly understood. Here, we isolated **human primary RPE** (hRPE) cells from 18 eye donors distributed over a wide age range (10–67 years old). A quantitative proteomic analysis was performed to analyze changes in their intracellular and secreted proteins. Age-group related subtypes and age-associated proteins were revealed and potential age-associated mechanisms were validated in ARPE-19 and hRPE cells. The results of proteomic data analysis and verifications suggest that RNF123- and RNF149-related protein ubiquitination plays an important role in protecting hRPE cells from oxidative damage during aging. In older hRPE cells, apoptotic signaling-related pathways were up-regulated, and endoplasmic reticulum organization was down-regulated both in the intracellular and secreted proteomes. Our work paints a detailed molecular picture of hRPE cells during the aging process and provides new insights into the molecular characteristics of RPE during aging and under other related clinical retinal conditions.

## Introduction

Retinal degeneration (RD) is caused by the gradual destruction of retinal cells [1], among which age-related macular degeneration (AMD) has become a leading cause of irreversible vision loss in the elderly worldwide [2]. It is predicted that RD may progress concurrently with the aging population [3]. The retina comprises the multi-layered neural retina and monolayered retinal pigment epithelium (RPE). The RPE is located between the photoreceptors and the vascular choroid and forms a critical barrier between the retina and systemic circulation. Therefore, RPE performs many essential functions to nourish and support the neural retina, including the recycling of vitamin A, daily phagocytosis of photoreceptor outer segment tips, and maintenance of blood-retinal barrier. Evidence suggests that RPE dysfunction and atrophy precede many RDs, leading to neuroretina impairment that results in significant vision loss [4].

The pathogenesis of age-related RD is complex and multifactorial, and the corresponding research models are limited. Oxidative stress is believed to contribute to the pathogenesis of age-related RD [5]. Therefore, ample studies using oxidative damage agents, such as paraquat and hydrogen peroxide (H_2_O_2_), to analyze the oxidative stress response mechanisms of RPE have been reported [6], [7], [8]. Immortal cell lines such as ARPE-19 have been commonly used to study the human retina. However, these cell lines can differ significantly in some features relative to human primary RPE (hRPE) cells [9]. Induced pluripotent stem cell (iPSC)-derived and human embryonic stem cell (hESC)-derived RPE cell lines may be promising models to study RPE. They have been proven to exhibit behaviors similar to *in vivo* RPE and are being tested in clinical trials for RPE replacement [10], [11]. Several studies have used induced iPSC-derived RPE cells from control and AMD patients to investigate AMD pathogenesis [12], [13]. However, unlike damaged RPE in AMD patients, iPSC-derived RPE cells from these patients are in a relatively healthy status. hRPE cells are the ideal tool for studying of RPE aging, but the shortage of suitable donors and poor redifferentiation capacity limits their applications. Nevertheless, the role of hRPE in retina aging remains unknown, which is a key factor in the pathogenesis of age-related RD. RPE is mainly responsible for the secretion of mediators that participate in the functional integrity of the RPs and the vascular choroid. The dysregulation of proteins secreted by the RPE has been found to be involved in the development of age-related RDs, especially in the loss of vascular invasion and barrier function [14].

In this study, we explored the changes in the intracellular and secreted protein levels of hRPE cells obtained from donors with a wide age range. A quantitative mass spectrometry (MS)-based proteome analysis was performed to characterize those over- and under-represented proteins with age. Inferences about the molecular pathways affected by aging in hRPE cells can be drawn from our data. Our work provides a resource to study the impact of aging on hRPE and presents solid data for future research on RPE and related clinical retinal conditions.

## Results

### General overview of the workflow

**Figure 1:**
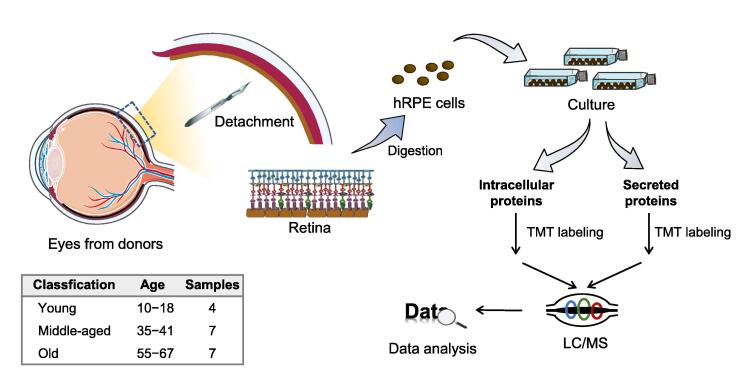
**Graphical illustration of study workflow** hRPE cells were isolated from donors’ eyes. After two passages, intracellular and secreted proteins of hRPE cells were extracted and analyzed by TMT10-based proteomic profiling. hRPE, human primary retinal pigment epithelium; TMT, tandem mass tag; LC/MS, liquid chromatograph-mass spectrometry.

An overview of the experimental strategy is presented in Figure 1. Eyes from male donors were obtained within 72 h after death from Henan eye bank (Zhengzhou, China). Previous medical and ocular histories were assessed to exclude donors with any eye disease. The eyes were subsequently divided into the following three groups according to donor ages: young (Y, 10–18 years, *n* = 4), middle-aged (M, 35–41 years, *n* = 7), and old (O, 55–67 years, *n* = 7) (Table S1). hRPE cells were immediately isolated and cultured for two passages. To confirm hRPE cell line, an immunofluorescence staining assay was performed using an RPE-specific antibody, anti-human RPE65 [15]. RPE65 is a cytosolic protein located around the 4′,6-diamidino-2-phenylindole (DAPI)-positive nucleus. Evidently, both hRPE and ARPE-19 cells showed similar morphology and RPE65 staining results (Figure S1A), indicating that cells collected from donor's eyes were RPE-derived cell lines.

To identify the changing patterns of intracellular and secreted proteome between the hRPE cell lines with different ages, we performed quantitative proteomics using TMT-10plex isobaric labeling reagents. At the third passage of hRPE cells, intracellular and secreted proteins were extracted and digested for further TMT labeling. To eliminate batch effects, samples were randomly labeled by TMT-10plex (Figure S1B). The mixture of intracellular (or secreted) protein samples was labeled as an internal standard in each batch of TMT-10plex and further analyzed by liquid chromatography tandem-MS analysis. A between-sample normalization using the median of intensities was performed before any quantification analysis (Figure S1C and D). Inferences about molecular pathways in hRPE cells affected by aging can be drawn from the data for over- and underrepresented proteins in older donors.

### Proteomic features of hRPE cells

**Figure 2:**
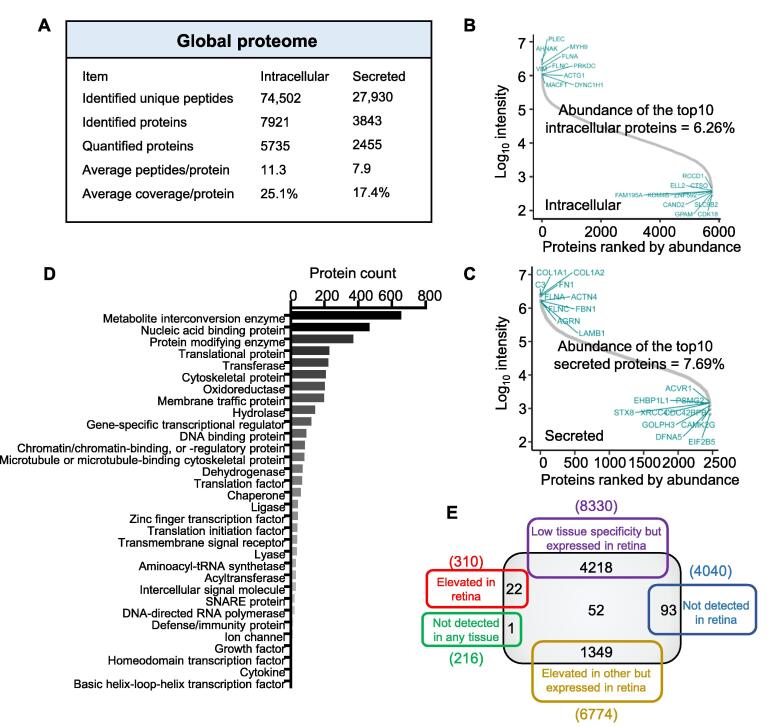
**Proteome overview of hRPE cells** **A.** The global proteome of hRPE cells. Protein abundance analysis of the quantified intracellular (**B**) and secreted (**C**) proteins. The X-axis indicates protein ranking orders according to their abundance. Y-axis represents the log_10_ intensity of each protein. The top 10 most abundant proteins are labeled in the top left. **D.** Numerical representations of indicated PANTHER protein categories of the quantified intracellular proteins. **E.** Venn diagram representing the numbers of expressed genes in the retina on mRNA level (the human protein atlas database-tissue atlas [retina]) and the quantified intracellular proteins in hRPE cells. The gray box represents intracellular quantified proteins in this study. PANTHER, protein analysis through evolutionary relationships.

In intracellular samples, we identified 74,502 unique peptides from 7921 proteins with an average sequence coverage of 25.1% [protein false discovery rate (FDR) < 1%]. Furthermore, 27,930 unique peptides were identified from 3843 secreted proteins with an average sequence coverage of 17.4% (FDR < 1%) (Figure 2A; Table S2). Among these identified proteins, 5735 intracellular proteins and 2455 secreted proteins were quantified in all samples (*n* = 18). Quality controls, including the number of quantified proteins and coefficient of variation (CV) of the Y, M, and O groups, were further analyzed. Results showed that the number of quantified proteins and the median CV values of the Y, M, and O groups were comparable (Figure S2A–D). The CV values were no more than 2.55% in intracellular samples and 3.22% in secreted samples. The top 10 most abundant intracellular proteins accounted for 6.26% of the total spectral abundance, including PLEC, AHNAK, MYH9, FLNA, VIM, FLNC, PRKDC, ACTG1, MACF1, DYNC1N1 (Figure 2B). Moreover, COL1A1, COL1A2, C3, FN1, FLNA, ACTN4, FLNC, FBN1, AGRN, and LAMB1 were the top 10 most abundant secreted proteins, accounting for 7.69% of the total abundance (Figure 2C). The aforementioned data indicate that protein quantification was robust.

To further characterize the properties of hRPE, we performed protein analysis through evolutionary relationships (PANTHER) protein class (version 15.0) and gene ontology (GO) subcellular location analysis. PANTHER protein class analysis indicated that the quantified intracellular proteins covered a broad range of protein classes, including metabolite inter-conversion enzyme (655 proteins), nucleic acid-binding protein (465 proteins), and protein modifying enzyme (369 proteins) (Figure 2D; Table S2). Subcellular location analysis revealed that most of the intracellular proteins belonged to the membrane (3157 proteins), nucleus (2895 proteins), and vesicle (1777 proteins) (Figure S2E; Table S2). As RPE cells are pigment-rich cells with obvious secretory properties [4], we also quantified 447 secretory vesicle proteins and 81 pigment granule proteins.

Subsequently, we matched intracellular proteins with the expressed genes in the retina on the mRNA level (the human protein atlas database-tissue atlas [retina]). Of the quantified proteins, the levels of 22 proteins were reported to be elevated in the retina than in other tissues. Besides, 93 proteins not detected in the retina and one protein not detected in any tissue were also quantified in our research (Figure 2E; Table S2). Next, those quantified secreted proteins were matched with the expressed genes of secreted proteins and vesicles on mRNA level, as predicted in the human protein atlas database-the cell atlas. Of the quantified proteins, 725 were predicted to be secreted proteins, whereas 302 belonged to vesicles (Figure S2F; Table S2). Therefore, our results provide broad coverage of the hRPE proteins in individuals.

### Variances in the hRPE proteomic profile during aging

**Figure 3:**
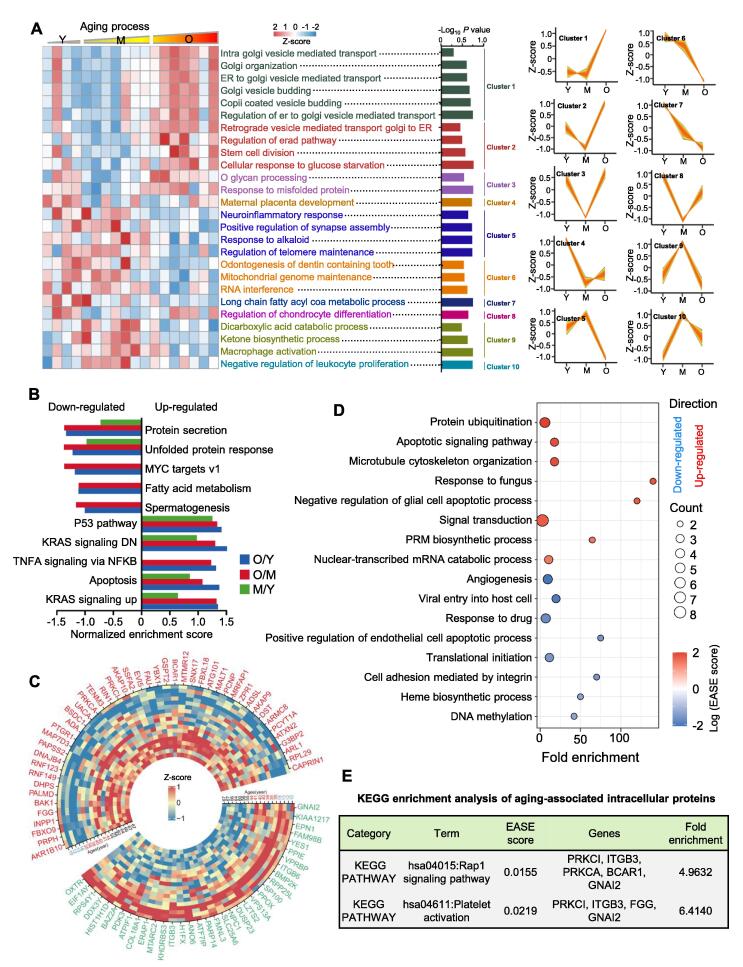
**Visualization of intracellular variation in proteins and their biological pathways** **A.** Significant discrete clusters to illustrate the relative expression changes of the intracellular proteomics. The heatmap of each sample (*n* = 18) for their subtype-associated molecular functions is shown on the left. Significant clusters shown on the right are clustered by mfuzz. **B.** GSEA analysis of the quantified proteins using hallmark database (version 7.0). GSEA was performed using version 4.0.3 of the GSEA desktop application. **C.** Heatmap of the age-associated proteins in hRPE cells. Age-associated proteins were defined as r > 0.5 (or < −0.5, protein intensities *vs*. ages) and *P* < 0.05 (Y *vs*. O). GO (biological process) (**D**) and KEGG (**E**) enrichment using DAVID (version 6.8) with the age-associated proteins. GSEA, gene set enrichment analysis; r, Pearson’s correlation coefficient; DAVID, the database for annotation, visualization, and integrated discovery; PRM, purine ribonucleoside monophosphate; EASE score, a modified Fisher exact *P* value; GO, gene ontology; KEGG, Kyoto encyclopedia of genes and genomes.

To illustrate the relationship between age groups and the related changes in molecular functions in hRPE, we employed protein clustering analysis to identify age group-related subtypes (Figure 3A; Table S3). In total, 10 clusters (C1–C10) were enriched with significant changes (*P* < 0.05) in molecular function. Apparently, those pathways enriched by proteins in C1, C5, and C6 are relatively stable in Y and M stages but change significantly in O. When entering the O stage, C1-related pathways such as intra-Golgi vesicle-mediated transport, COPII-coated vesicle budding, and Golgi vesicle budding were up-regulated. C5 and C6 related pathways, including neuroinflammatory response and the positive regulation of synapse assembly, were down-regulated in O. As indicated by C7, proteins enriched in long-chain fatty acyl CoA metabolic process were decreased during the aging process. Therefore, pathways enriched by proteins in C1, C5, C6, and C7 may be closely related to the pathological process of age-related RD. However, compared with O and Y, proteins in the M stage exhibited down-regulated expression levels in pathways enriched by proteins in C2, C3, and C8, including cellular response to glucose starvation (C2), response to misfolded protein (C3), and regulation of chondrocyte differentiation (C8). Besides, proteins in the M stage exhibited specific up-regulation in C9 and C10, such as macrophage activation (C9) and negative regulation of leukocyte proliferation (C10).

To further analyze the relationships between different stages (O *vs*. Y, M *vs*. Y, and O *vs*. M) and reveal the influences of the aging process on hRPE, proteomic data were analyzed by gene set enrichment analysis (GSEA) (hallmark database, version 7.0). It was found that O/Y and O/M exhibited similar changing patterns in most enriching hallmarks (Figure 3B; Table S3). This phenomenon suggested that these proteins underwent noticeable changes when approaching the O stage, which might be the reason that RPE-related diseases were prone to morbidity in old age [3]. Besides, during the aging process, hRPE cells were down-regulated in some processes (such as protein secretion and unfolded protein response) and up-regulated in others (such as the p53 pathway and apoptosis). The aforementioned hallmark changes may contribute to the pathogenesis of RPE aging.

### Age-associated proteins and related functions

To identify age-associated proteins in hRPE cells, we calculated Pearson’s correlation coefficient (r) for the intensities of the quantified proteins between the different age groups. The cut-off value for r was defined as 0.5, and *P* value (Y *vs*. O) was set to <0.05. In total, 46 proteins (such as AKR1B10, PRPH, and FBXO9) were found to be up-regulated, whereas 35 proteins (such as OXTR, EIF1AY, and RPS4Y1) were down-regulated (Figure 3C; Table S4). In particular, the up-regulated AKR1B10 was reported to exert a protective role by eliminating oxidative stress and was identified as a biomarker in older hepatocellular carcinoma patients [16], [17].

To better demonstrate the functional information of age-related proteins, we performed GO and Kyoto encyclopedia of genes and genomes (KEGG) enrichment analyses of these proteins using the database for annotation, visualization, and integrated discovery (DAVID, version 6.8). GO analysis showed that down-regulated proteins, including FMNL3, COL18A1, ATPIF1, and ERAP1, might contribute to angiogenesis, a pathological hallmark in many retinal vascular diseases [18] (Figure 3D; Table S4). Therefore, the four down-regulated proteins might be associated with the pathogenesis of these diseases. Besides, five up-regulated proteins, including RNF149, RNF123, FBXO9, PCNP, and MALT1, were found to be involved in protein ubiquitination (Figure 3D; Table S4). Ubiquitination-mediated protein degradation is the selective degradation of damaged proteins. The five ubiquitination-related proteins may counteract the insults accumulated in proteins by removing damaged parts during aging, especially RNF149 and RNF123, two E3 ubiquitin ligases [19], [20]. As presented in Figure 3E, the up-regulated proteins PRKCA, PRKCI, BCAR1, GNAI2, and ITGB3 belong to the RAP1 signaling pathway (Table S4). RAP1 signaling pathway has previously been reported to protect the telomeres of senescent cells from DNA damage [21]; thus, these five proteins may be contributing to the DNA damage observed during aging.

### RNF123, RNF149, and protein ubiquitination may protect RPE from oxidative damage during aging

**Figure 4:**
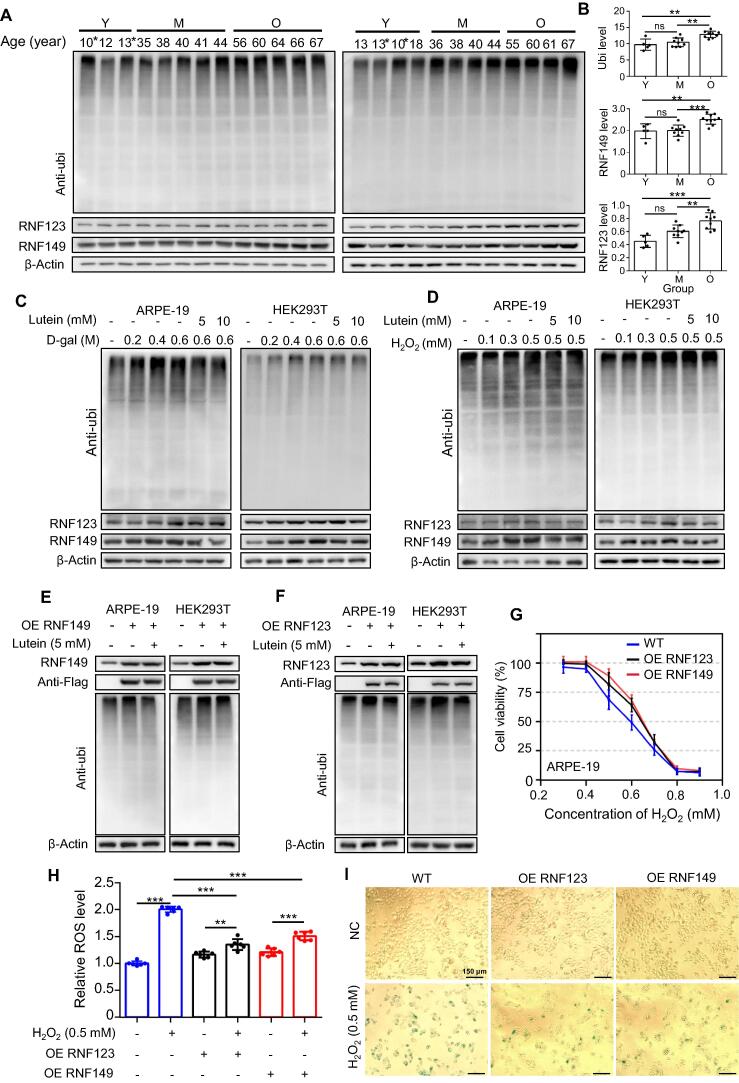
**Functional analysis of RNF123, RNF149, and protein ubiquitination** **A.** WB image of the hRPE cells with different ages. The noted age represents the age of the hRPE donor. **B.** Relative grey date of protein ubiquitination (up), RNF149 (middle), and RNF123 (low) levels in different ages. Samples marked with an asterisk in (A, left) and (A, right) are the same samples, namely 10-year-old and 13-year-old young samples. These two samples were used as internal standards to normalize grayscale differences between the two images. **C.** WB analysis of the ARPE-19 (left) and HEK293T (right) cells stimulated with gradient concentrations of D-gal for 4 h. **D.** WB analysis of the ARPE-19 (left) and HEK293T (right) cells stimulated with gradient concentrations of H_2_O_2_ for 30 min. RNF123, RNF149, and ubiquitinated proteins were up-regulated in ARPE-19 cells with increasing concentrations of H_2_O_2_, and this phenomenon can be partially reversed by lutein. WB analysis of ARPE-19 (left) and HEK293T (right) cells overexpressing RNF149 (**E**) or RNF123 (**F**). RNF123 or RNF149 overexpressing significantly increased levels of protein ubiquitination in ARPE-19 and HEK293T cells, and this phenomenon could be partially reversed by lutein. **G.** Viability analysis of ARPE-19 cells treated with H_2_O_2_. ARPE-19 cells became less sensitive to H_2_O_2_ after RNF123 or RNF149 overexpression. **H.** ROS production in ARPE-19 cells treated with H_2_O_2_. H_2_O_2_-induced ROS generation was significantly inhibited by RNF123 or RNF149 overexpression. **I.** SA-β-gal activity in ARPE-19 cells. Scale bar, 150 µm. WB results were quantified thrice using ImageJ software (version 1.52a, NIH, USA) and presented as the ratio of target protein grey value to β-actin grey value. WB, Western blotting; OE, overexpression; ROS, reactive oxygen species; SA-β-gal, senescence-associated β-galactosidase; anti-ubi, anti-ubiquitination; NC, normal control; WT, wild type; ns, no significance; *, *P* < 0.05; **, *P* < 0.01; ***, *P* < 0.005.

To demonstrate the reliability of our proteomic data and reveal the relationships between RNF123, RNF149, the ubiquitinated proteins, and aging, we performed Western blotting (WB). We found that the levels of RNF123, RNF149, and ubiquitinated proteins increased with age (Figure 4A and B). Oxidative stress is a hallmark of aging; previous studies have used D-galactose (D-gal)-treated or H_2_O_2_-treated cells to analyze aging mechanisms [22], [23]. To verify the effect of RNF123, RNF149, and the related protein ubiquitination in RPE aging process, ARPE-19 and HEK293T cells with gradient concentrations of oxidative damage inducer (H_2_O_2_ and D-gal) treatment were analyzed. WB results showed that the levels of RNF123, RNF149, and ubiquitinated proteins increased with increasing concentrations of D-gal (or H_2_O_2_). However, this phenomenon can be reversed by lutein, a natural antioxidant (Figure 4C and D, Figure S3A–D). Next, hRPE cells from Y and O donors were further treated with gradient concentrations of H_2_O_2_. CCK8 viability results showed that hRPE cells from O donors were more vulnerable to oxidative damage (H_2_O_2_) than those from Y donors (Figure S3E). WB results indicated that oxidative damage was related to the up-regulation of protein ubiquitylation, which can be rescued by lutein as well (Figure S3F). To explore the relationships between RNF123 or RNF149 and protein ubiquitination levels, RNF123 and RNF149 were overexpressed in ARPE-19 and HEK293T cells. Results revealed that RNF123 or RNF149 overexpression significantly increased protein ubiquitination levels in ARPE-19 and HEK293T cells; this phenomenon could be partially reversed by lutein (Figure 4E and F, Figure S3G and H). To further confirm the functions of RNF149 or RNF123 in RPE cells, we studied the tolerance of ARPE-19 cells to oxidative damage after RNF123 or RNF149 overexpression. Viability analysis showed that following RNF123 or RNF149 overexpression, ARPE-19 cells became less sensitive to H_2_O_2_ (Figure 4G). The accumulation of reactive oxygen species (ROS) is an indicator of oxidative damage in cells. Therefore, we examined ROS generation in ARPE-19 cells treated with H_2_O_2_ after RNF123 or RNF149 overexpression. As shown in Figure 4H, ROS levels were significantly up-regulated in ARPE-19 cells treated with H_2_O_2_, but these levels were significantly decreased after RNF123 or RNF149 overexpression. In other words, H_2_O_2_-induced ROS generation was significantly inhibited by RNF123 or RNF149 overexpression in ARPE-19 cells. We subsequently achieved the same results in HEK293T cells (Figure S3I). Senescence-associated β-galactosidase (SA-β-gal) is an important marker of aging cells. We further studied the roles of RNF123 and RNF149 in oxidative damage-related aging cells. We detected changes in SA-β-gal levels in ARPE-19 cells treated with H_2_O_2_ following RNF123 or RNF149 overexpression. As shown in Figure 4I, SA-β-gal levels increased significantly in H_2_O_2_-treated cells. Conversely, in cells treated with H_2_O_2_, SA-β-gal levels decreased after RNF123 or RNF149 overexpression. The aforementioned results suggest that RNF123 or RNF149 overexpression could reduce the oxidative damage in cells during aging, which was related to the changes in ubiquitination levels. Therefore, RNF123, RNF149, and related protein ubiquitination may play important roles in protecting RPE from oxidative damage.

### Age-associated secretory phenotype and proteins

**Figure 5:**
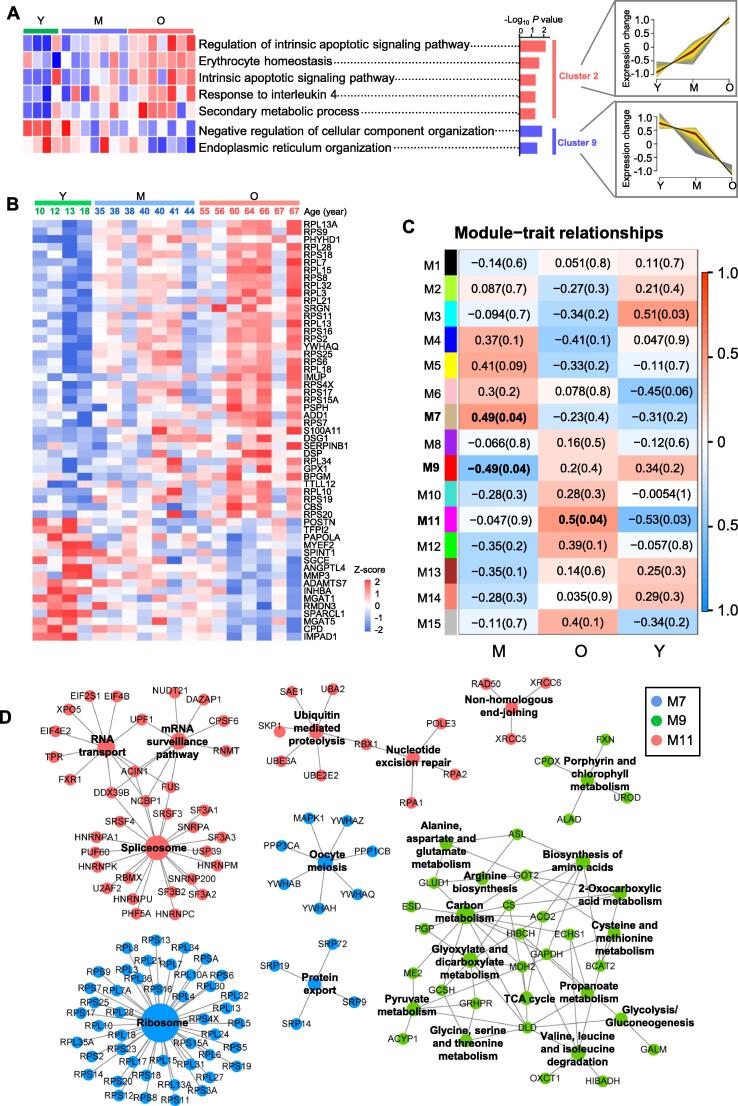
**Visualization of age-associated secretory phenotype** **A.** Significantly down- and up-regulated clusters to illustrate the relative expression changes of the secreted proteins. The heatmap of each sample (*n* = 18) for their subtype-associated molecular functions is shown on the left. Clusters shown on the right are the up- and down-regulated clusters that were clustered by mfuzz. **B.** Heatmap of the age-associated secreted proteins. Age-associated secreted proteins were defined as r > 0.5 (or < −0.5, protein intensities *vs*. ages), and *P* < 0.05 (Y *vs*. O). **C.** Aging-relevant protein modules associated with age traits. Correlation coefficients are indicated on the left with corresponding *P* values in round brackets on the right. Significant correlations (*P* < 0.05) are labeled in bold text. **D.** KEGG network depiction of protein co-expression modules. Edges (lines) represent connections between the nodes, and nodes indicate proteins. Blue represents M7 (positive correlations), green represents M9 (negative correlations), and red represents M11 (positive correlations).

Mediators secreted by RPE are involved in the functional integrity of the RPs and the vascular choroid [14]. To determine age-associated secretory phenotype, we performed a clustering analysis of the secreted proteins. In total, nine clusters (C1–C9) were enriched (Figure 5A, Figure S4A; Table S5). Apparently, pathways enriched by proteins in C2 were up-regulated with age. These pathways were related to apoptotic signaling pathways, including the regulation of intrinsic apoptotic signaling. The up-regulation of apoptotic signaling-related pathways was consistent with intracellular GSEA analysis, including the up-regulation of proteins in the p53 pathway and apoptosis (Figure 3D). However, pathways such as those negatively regulating cellular component organization and endoplasmic reticulum (ER) organization enriched by proteins in C9 were down-regulated with age. ER is the entry site for proteins into the secretory pathway [24]. Therefore, the down-regulated ER organization in the secretome was consistent with the intracellular results that proteins in protein secretion and unfolded protein response were down-regulated in hRPE during aging.

Age-associated secreted proteins were further analyzed using *P* < 0.05 (Y *vs*. O) and *r* > 0.5 (or *r* < −0.5, protein intensities among different age groups). As shown in Figure 5B, 38 proteins (including SRGN and DIS3) were determined to be up-regulated, whereas 16 proteins (including MYEF2 and SPARCL1) were down-regulated. Many of the age-associated secreted proteins have been reported to be associated with age-related diseases or as age-related proteins. For example, the increased expression of SRGN has been confirmed to be associated with greater aggressiveness in inflammation [25], [26], and SPARCL1 has been reported to be enriched in serum from young mice [27].

### Secreted protein network alterations associated with aging

To gain system-level insights into age-associated secretome alterations of hRPE, we performed protein co-expression network analysis using weighted gene co-expression network analysis (WGCNA). In total, 15 strongly co-expressed protein network modules were identified (Figure 5C; Table S5). These modules were color-coded according to the convention of WGCNA and then labeled as M1–M15 (Figure S4B). To identify age-relevant modules, the module-trait relationships determined by biweight midcorrelations between each module eigenprotein and sample variables were assessed. Three modules that were significantly correlated (*P* < 0.05) with age were identified, including two positively correlated modules (M7 and M11) and one negatively correlated module (M9).

To get insights into the biological roles of age-associated modules, the molecular and functional characteristics of these 3 modules were further analyzed based on KEGG and GO databases. KEGG enrichment analysis revealed that positively correlated modules (M7 and M11) were significantly enriched (correct *P* value < 0.05) in RNA-related pathways (including RNA transport, mRNA surveillance pathway, and spliceosome), ubiquitin-mediated proteolysis, nucleotide excision repair, non-homologous end-joining, ribosome, oocyte meiosis, and protein export (Figure 5D). Particularly, ubiquitin-mediated proteolysis enriched by secreted proteins including SAE1, UBA2, RBX1, UBE2E2, UBE3A, and SKP1, was also defined as an age-associated functional change in intracellular proteomics analysis (Figure 3D), which was further verified by WB (Figure 4A and B). In addition, pathways related to small molecular biosynthesis or metabolism were enriched in negatively correlated modules (M9), such as alanine, aspartate, and glutamate metabolism; arginine biosynthesis; and amino acid biosynthesis. GO enrichment analysis indicated that the categories of the structural constituent of ribosome (M7), translation regulator activity (M11), processes linked to the binding (M7 and M11, including ribonucleoprotein complex binding, cadherin binding, single-stranded DNA binding, cell adhesion molecule binding, rRNA binding) were up-regulated with age (Figure S4C; Table S5). However, enzymatic activity (including hydro-lyase activity, carbon–oxygen lyase activity, and lyase activity), NAD binding, and coenzyme binding were down-regulated with age.

### Specifically changed secreted proteins in hRPE cells during aging

**Figure 6:**
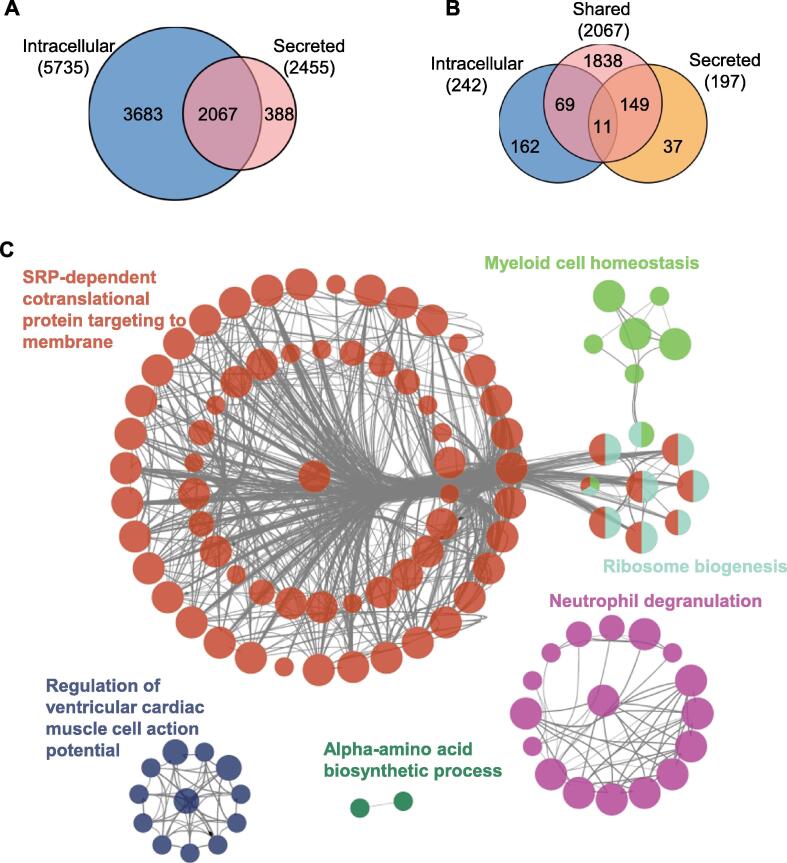
**Visualization of the specifically changed secreted proteins** **A.** Venn diagram depicting the overlap between the quantified intracellular (blue) and secreted (red) proteins. **B.** Venn diagram representing the overlap of significantly regulated intracellular (blue) and secreted (yellow) proteins versus the shared proteins of Venn diagram (red). **C.** Functional enrichment analysis of the specifically changed secreted proteins. Analysis was performed using ClueGo software (version 2.5.7) in Cytoscape (version 3.7.2).

Many proteins are present both inside the cell and within the extracellular space, and changes specifically in secreted proteins may be essential for intercellular communication during aging. Therefore, proteins quantified both in intracellular and secreted hRPE samples were further analyzed. In total, 2067 proteins were shared by intracellular and secreted samples, and 147 of them were significantly changed (*P* < 0.05) in secreted proteins, including IMPAD1, CPD, RMDN3, MGAT1, MYEF2, and PAPOLA (Figure 6A and B; Table S6)*.* Functional enrichment analysis of the specifically changed secreted proteins was performed by ClueGo software (version 2.5.7) in Cytoscape (version 3.7.2). The six clusters of 108 significantly enriched GO terms (biological processes) were identified, including SRP-dependent co-translational protein targeting to membrane, myeloid cell homeostasis, regulation of ventricular cardiac muscle cell action potential, neutrophil degranulation and ribosome biogenesis, and alpha-amino acid biosynthetic process (Figure 6C). These biological processes may play an important role in intercellular communication in RPE aging.

## Discussion

RPE performs many essential functions to nourish and support the neural retina and is of vital importance in the pathogenesis of age-related RD, such as AMD [28]. However, the exact molecular changes in the RPE during aging that mediate dysfunction remain poorly understood. Ample studies using immortal cell lines (such as ARPE-19) or iPSC- or hESC-derived RPE cell lines to explore the pathology of RPE-related disease have been reported [6], [7], [12], [13]. Owing to the restricted sources of human RPE cells, limited pathology studies with human RPE have been performed. In this study, we collected hRPE cells from 18 eye donors distributed over a wide age range (10–67 years) to analyze the molecular changes in RPE during aging. And the cells were further confirmed by an RPE-specific antibody (anti-human RPE65).

Quantitative MS-based proteome analyses with isobaric labeling reagents such as TMT have been widely used to profile the changes in protein levels among different samples [29]. In this work, we used an MS-based quantitative approach for proteome and secretome analysis, which provided broad coverage of the proteins of hRPE over a wide age range. The biological function of most of the proteins reported in this study was deduced based on an extensive literature review, rather than only relying on Uniprot or GO database annotation. We believe that our approach and data are robust and can produce a descriptive quantitative dataset to present aging-related molecular alternations. An important limitation of this work is that proteomic analysis provides a static image of the protein concentration at one point in time. Therefore, we did not present information on the dynamics of protein accumulation (or loss) or their post-translational modification (*e.g.*, phosphorylation and acetylation). Addressing these important parameters will help us to better interpret the biological changes more fully.

Herein, we described the proteome and secretome features of hRPE cells that have not been previously described. Furthermore, we revealed the variations in hRPE during aging and discovered the age-associated proteins and their related functions. GSEA analysis of the intracellular proteins indicated that hRPE cells were down-regulated during protein secretion and unfolded protein response but up-regulated in the p53 pathway and apoptosis. Besides, altered secretory functions such as the down-regulation of ER organization and up-regulation of apoptotic signaling-related pathways were consistent with the intracellular changes. Cells will become more vulnerable to environmental damage in aging. ER is the site of entry for proteins into the secretory pathway [24]. Unfolded protein response allows the cell to manage ER stress that is imposed by the secretory demands associated with environmental damage [30]. p53 has been reported to be involved in DNA repair, apoptosis, and cellular stress responses and also plays an important role in modulating cellular senescence and organismal aging [31]. Therefore, molecular changes like down-regulation in protein secretion and unfolded protein response together with up-regulation in the p53 pathway and apoptosis may contribute to the pathogenesis of RPE aging. RNF149 and RNF123 are two E3 ubiquitin-protein ligases that regulate protein degradation through their ubiquitination [21]. WB results in our study showed that RNF123, RNF149, and the ubiquitinated proteins were up-regulated with the aging process of hRPE. The functional analysis of cells overexpressing RNF123 and RNF149 revealed that RNF123, RNF149, and related protein ubiquitination may play an important role in protecting RPE from oxidative damage.

Regarding the proteome properties of RPE-related cells, it has been reported that ARPE-19, hESC-RPE, and hRPE cells exhibit a high degree of similarity in proteomes [32], [33]. However, hRPE cells expressed a large number of metabolic, transport, and mitochondrial proteins [33]. PANTHER protein class analysis in our study indicated that the number of proteins in metabolite inter-conversion enzyme is the largest (Figure 2D). Pelkonen et al. quantified 16 transporter proteins in ARPE-19 and human fetal RPE (hfRPE) cells using quantitative targeted absolute proteomics [34]. In our research, 86 transporter proteins were quantified in all of the 18 samples; among these, 8 transporters have been previously quantified in hfRPE and ARPE-19 cells [34]. In AMD-related proteomics analysis, Nordgaard et al. analyzed the proteome changes of hRPE cells from four donor eyes during the progressive stages of AMD. They revealed that proteins involved in protecting from stress-induced protein unfolding and aggregation, apoptosis regulation, and mitochondrial trafficking and refolding changed early in AMD progression [35]. Proteins associated with anti-proliferation, apoptosis induction, and oxidative-stress protection underwent changes after ultraviolet radiation irradiation in ARPE-19 cells [36]. Likewise, in our research, unfolded protein response was down-regulated, and apoptosis-related pathways were up-regulated (Figure 3B). In addition, we also identified that RNF123- and RNF149-related protein ubiquitination may play an important role in protecting from oxidative damage during aging. Alcazar et al. investigated the proteomic profile of RPE cell membrane blebs induced by hydroquinone in ARPE-19 cells. Their results indicated that cell adhesion may be involved in AMD progression [37]. ARPE-19 cell proteins involved in transport and ECM remodeling have been reported to be altered with cigarette smoke treatment [34]. Besides, paraquat will change the expressions of proteins related to focal adhesion, TGFβ signaling, and ECM. Our study also revealed the aging-associated proteins related to the regulation of cell adhesion (*e.g.*, PRKCA, MALT1, and TENM3), immune response-activating signal transduction (*e.g.*, ADA, FGG, and BCAR1), and ECM organization (*e.g.*, ITGB3, ITGB6, and COL18A1). However, MMP-14, which was identified in RPE blebs, did not show a significant age correlation in our results [37]. It is considered that ROS will increase glycolytic protein levels but decrease mitochondrial complex I subunit levels; however, the levels of neither of these proteins change significantly in our work [38]. The aforementioned phenomenon may be attributed to some differences between AMD-related damage models and the natural aging model of hRPE from healthy donors in our work.

In conclusion, our results paint a detailed molecular picture of human RPE in the aging process and present solid data that will aid in future research on RPE and related retinal conditions.

## Materials and methods

### Cell culture

ARPE-19 cells were purchased from the American type culture collection (ATCC) (Catalog No. CRL-2302, ATCC, Manassas, VA). HEK293T cells were from the ATCC (Catalog No. CRL-3216, ATCC, Manassas, VA). hRPE cells were obtained from the eyes of donors (Henan eye bank) according to previously published protocols [39]. Briefly, the eyes were cut circumferentially above the equator, and the lens, vitreous, and iris tissue were removed. The choroid was separated from the sclera. hRPE cells were removed from the choroid by incubation in 7.5% trypsin-EDTA solution. Cells were grown in a mixture (1:1) of Dulbecco’s modified Eagle’s medium and nutrient F-12 Ham (Catalog No. C11995-065, Gibco, Carlsbad, CA) supplemented with 10% fetal bovine serum (Catalog No. SFBE, NATOCOR, Córdoba, Argentina), 100 U/ml penicillin and 100 U/ml streptomycin (Catalog No. 15140-122, Gibco, Carlsbad, CA) and were cultured in 37 °C with 5% CO_2_ incubator.

### Compounds and antibodies

The antibodies and compounds used were commercially obtained: anti-RPE65 antibody (Catalog No. ab13826, Abcam, Cambridge, MA), anti-RNF123 antibody (Catalog No. A09642-1, BOSTER, China), anti-RNF149 (Catalog No. bs-9228R, Bioss, China), anti-ubiquitin antibody (Catalog No. 3933, Cell Signaling Technology, Boston, MA), anti-β-Actin (Catalog No. EM21002, Huabio, China), D-galactose (D-gal, Catalog No. G0750, Sigma-Aldrich, St. Louis, MI), H_2_O_2_ (Catalog No. 323381, Sigma-Aldrich), DAPI (Catalog No. 9542, Sigma-Aldrich), and lutein (Catalog No. 07168, Sigma-Aldrich).

### Cell transfection

The coding sequences of RNF123 (NM_022064.5) and RNF149 (NM_173647.7) containing an N-terminal FLAG-tag were synthesized by cloning into the pcDNA3.1 vector. All constructs were verified through Sanger sequencing (Sangon Biotech, China). The cells were grown in a 10 cm culture dish until 70% confluency was reached and then transfected with Lipofectamine 3000 (Catalog No. L3000015, Invitrogen, Carlsbad, CA) according to the manufacturer’s instructions.

### Cell viability assay

The effects of H_2_O_2_ on the viability of hRPE and ARPE-19 cells were explored by cell counting kit-8 (CCK-8, Catalog No. 96992, Sigma-Aldrich) according to the manufacturer’s instructions. Briefly, approximately 4000 hRPE or ARPE-19 cells were seeded in 96-well plates and allowed to adhere for 12 h. Next, gradient concentrations of H_2_O_2_ (or supplement with 0, 5, or 10 mM lutein) were added and incubated with the cells for another 48 h before WST-8 addition to each well. After incubation for another 1 h, absorbance was measured using a multifunction microplate reader (PerkinElmer, Waltham, MA). hRPE or ARPE-19 cells cultured without any treatment were used as control.

### Immunofluorescence staining

hRPE and ARPE-19 cells were seeded in 12-well plates and cultured to 80% confluence. All cells were subsequently fixed with 4% paraformaldehyde (Catalog No. G27810, Mengbio, China) for 30 min at 25 °C, washed with phosphate buffered saline (PBS, pH 7.4) thrice, permeabilized with 0.2% Triton X-100 (Catalog No. 36090, Dowobio, China) for 20 min, and washed with PBS thrice. The cells were then blocked in PBS containing 5% BSA for 2 h and incubated with anti-RPE65 antibody overnight at 4 °C. Following incubation, cells were washed thrice with PBS and then incubated with fluorescent-conjugated secondary antibody (Catalog No. 4408, Cell Signalling Technology, Boston, MA) for 1.5 h at room temperature, followed by 5 min of nuclear staining with DAPI. Fluorescent images were taken by using a fluorescence microscope (OLYMPUS IX73, Tokyo, Japan).

### ROS measurements

ROS levels were detected by the ROS assay kit (Catalog No. S0033, Beyotime Biotechnology, China) according to the manufacturer’s instructions. Approximately 6000 ARPE-19 cells (or those overexpressing RNF123 or RNF 149) were seeded in 96-well plates and allowed to adhere for 24 h. H_2_O_2_ in gradient concentrations was subsequently added and incubated with the cells for another 4 h before analysis.

### SA-β-gal measurements

The cellular senescence of H_2_O_2_-treated ARPE-19 cells was examined using a β-Galactosidase detection kit (Catalog No. C0602, Beyotime Biotechnology, China) according to the manufacturer’s instructions. Approximately 3 × 10^5^ ARPE-19 cells (or those overexpressing RNF123 or RNF 149) were seeded in 6-well plates. After 24 h, H_2_O_2_ was added in gradient concentrations and incubated with the cells for another 12 h before analysis.

### Oxidative damage cell model for verification

ARPE-19 or HEK293T cells were grown in a 10 cm culture dish until 80% confluency and then treated with gradient concentrations of lutein (0, 5, and 10 mM) for 6 h. Subsequently, the cell culture medium was exchanged with gradient concentrations of H_2_O_2_ for 30 min before analysis. For D-gal treatment, the cell culture medium was exchanged with gradient concentrations of D-gal for 4 h before analysis.

### Intracellular and secreted protein lysate preparation

For secreted proteins, hRPE cells were washed thrice with PBS (pH 7.4) before adding 5 ml of RPE media (without serum). The media was collected after culturing cells for 24 h, and the secretome was washed and concentrated using a centrifugal ultrafiltration membrane (molecular weight cutoff 15 kDa, Merck Millipore Ltd, Darmstadt, Germany). For intracellular proteins, hRPE cells were washed twice with ice-cold PBS, harvested with a cell scraper, and collected by low-speed centrifugation. Then both the secretome (for secreted proteins) and hRPE cells (for intracellular proteins) were lysed with RIPA buffer containing protease inhibitor cocktail [1% NP-40, 0.5% (w/v) sodium deoxycholate, 150 mM NaCl, and 50 mM Tris (pH 7.5)], followed by 3 min sonication [3 s on and 10 s off with 195 watt of JY92-IIN (NingBoXinYi, China)]. Protein quantification was performed by Bradford assay (Catalog No. 5000205, Bio-Rad, Hercules, CA).

### Proteomic sample preparation

Each protein sample was diluted to 1 μg/μl (50 μg for intracellular protein analysis and 20 μg for secreted protein analysis) by 100 mM tetraethylammonium bromide (TEAB, Catalog No. 15715-58-9, Sigma-Aldrich); reduced by Tris (2-carboxyethyl) phosphine (TCEP, Catalog No. C4706, Sigma-Aldrich) with a final concentration of 10 mM at 56 °C for 1 h; alkylated with iodoacetamide (IAA, Catalog No. I1149, Sigma-Aldrich) with a final concentration of 20 mM in the dark at room temperature for 30 min; precipitated with methanol, chloroform, and water (CH_3_OH:CHCl_3_:H_2_O = 4:1:3); followed by overnight digestion with trypsin (Catalog No. V5117, Progema, Madison, WI; 1:50 enzyme to protein). The tryptic peptides of each sample were labeled with TMT-10plex (Catalog No. 90113CH, ThermoFisher Scientific, Waltham, MA) reagents according to the manufacturer’s protocol. One batch of 10-plex TMT kits was used to label nine intracellular (or secreted) samples and one mixture of hRPE intracellular proteins (or secreted proteins). After quenching with 5% hydroxylamine, TMT-labeled peptides of each batch of TMT-10plex were mixed and desalted by using a C18 column. The desalted peptides were subsequently fractionated by Agilent Poroshell HPH C18 column (250 mm × 4.6 mm, OD 4 μm) on Agilent 1260 instrument. Buffer A (2% ACN, 10 mM NH_4_COOH, pH 10) and a non-linear increasing concentration of buffer B (90% ACN, 10 mM NH_4_COOH, pH 10) were used for peptide separation at 1 ml/min. A standard 120 min gradient was used as follows: 0%−8% B for 10 min, 8%−35% B for 70 min, 35%−60% B for 15 min; 60%−70% B for 10 min, and 70%−100% B for 15 min. The peptide mixtures were separated into 120 fractions and combined by a concatenation strategy into 24 fractions (1&25&49&73&97, …, 24&48&72&96&120). The combined fractions were used for further liquid chromatograph-mass spectrometer/mass spectrometer (LC-MS/MS) analysis.

### LC-MS/MS analysis

Peptides were dissolved in buffer A (0.1% formic acid, FA) and then directly loaded onto a reversed-phase analytical column (Acclaim PepMap RSLC, Thermo, Waltham, MA). The applied gradient for solvent B (0.1% FA in 98% CH_3_CN) increased from 6% to 22% for 42 min, 22% to 30% for 12 min, climbing to 80% in 3 min and then holding at 80% for the last 3 min, all at a constant flow rate of 500 nl/min on an EASY-nLC 1000 ultra performance liquid chromatography (UPLC) system. The peptides were then subjected to a nanospray ionization source followed by tandem MS (MS/MS) in Orbitrap Fusion Lumos instrument (ThermoFisher Scientific, Waltham, MA,) coupled online to the UPLC. Intact peptides were detected in the Orbitrap at a resolution of 60,000. Peptides were selected for MS/MS using a normalized collision energy setting as 32, and ion fragments were detected in the Orbitrap at a resolution of 15,000. A data-dependent procedure that alternated between one MS scans and 20 MS/MS scans was applied for top 20 precursor ions above a threshold intensity > 1 × 10^4^ in the MS survey scan with 30.0 s dynamic exclusion. The electrospray voltage applied was 2.4 kV. To prevent overfilling of the orbitrap, automatic gain control was used. 5 × 10^4^ ions were accumulated for MS/MS spectra generation. For MS scans, the *m/z* scan range was 350–1550. The fixed first mass was set as 100 *m*/*z*.

### MS data processing

Raw MS/MS data were processed by MaxQuant with an integrated Andromeda search engine (version 1.5.2.8). Tandem mass spectra were searched against the human UniProt database (20,239 entries, 2017/09) concatenated with the reverse decoy database. Trypsin/P was set as a cleavage enzyme. The missing cleavages were set as 2. The mass error was set to 0.02 Da for fragment ions and 10 ppm for precursor ions. Carbamidomethylation on cysteine was the fixed modification. Acetylation on protein N-terminal and oxidation on methionine were specified as variable modifications. The minimum peptide length was set at 7. FDR thresholds for modification site, peptide, and protein were specified at 1%. TMT-10plex was selected as the quantification method. All the other parameters in MaxQuant were set as default values. Intensities were extracted and normalized based on their total intensity and the internal standard to correct the sample loading difference. The zero intensity proteins, reverse, and contaminant were excluded.

### Bioinformatics analysis

Clustering analysis was performed using ‘mfuzz’ (R package). Pathway analysis for age-related intracellular proteins was performed through the database for annotation, visualization and integrated discovery (DAVID) bioinformatics resources (version 6.8) [40]. GSEA was conducted using tumour hallmark database (version h.all.v7.0.symbols.gmt [hallmarks]) [41]. Functional enrichment analysis for determining the biological relevance of the hub genes and their protein regulators specifically changed in the secretome was performed using Cytoscape (version 3.7.2) plugged with ClueGO (version 2.5.7) [42].

### Comparison between mRNA levels of the expressed genes

Transcriptome data of the retina were obtained from the human protein atlas database-tissue atlas website (https://www.proteinatlas.org/humanproteome/tissue/retina). Data in ‘the distribution of all genes across five categories based on transcript specificity in the retina as well as in all other tissues’ were downloaded (namely, ‘elevated in the retina [310genes]’, ‘not detected in any tissue [216genes]’, ‘not detected in the retina [4040genes]’, ‘low tissue specificity but expressed in retina [8330genes]’, and ‘elevated in other but expressed in retina [6774genes]’). Genes in the aforementioned five categories were overlapped with the genes encoding quantified intracellular proteins in our work. The expressed genes of secreted proteins and vesicles on mRNA level were acquired from the human protein atlas database-the cell atlas (https://www.proteinatlas.org/humanproteome/cell). In particular, data in ‘secretory-secreted proteins’ and ‘secretory-vesicles’ were downloaded and overlapped with the genes encoding the quantified secreted proteins in our work.

### Protein co-expression network analysis

Protein co-expression network analysis was performed with the R package WGCNA using the entire secreted proteomic data set of all quantified proteins according to a previous report [43]. The eigengene value of each module was calculated to test the correlation with each sample [44]. Before creating the network model, the distribution of the entire dataset was performed. The hub genes from the model were screened by calculating the connectivity degree of each gene with Cytoscape (version 3.7.2) [45].

### WB analysis

Protein extracts was separated on 10% sodium dodecyl sulfate–polyacrylamide gel electrophoresis and then transferred onto a polyvinylidene difluoride membrane (Millipore, Burlington, MA). After blocking with 5% milk solution in Tris-buffered saline with Tween (TBST) for 1 h, membranes were incubated with 3% milk containing appropriate primary antibodies overnight at 4 °C, followed by 2 h of incubation with horseradish peroxidase-conjugated secondary antibodies. Target protein band signals were detected using a chemiluminescent detection reagent.

## Ethical statement

This study has been approved by the medical ethics committee of Henan provincial eye hospital (Approval No. HNEEC-2020 [14]). And the written informed consent has been obtained from the participating subjects.

## Data availability

The mass spectrometry raw data and the corresponding txt files have been deposited in the ProteomeXchange consortium via the iProX partner repository [46] (ProteomeXchange: PXD028202), which are publicly accessible at http://proteomecentral.proteomexchange.org.

## CRediT author statement

**Xiuxiu Jin:** Conceptualization, Methodology, Resources, Validation, Formal analysis, Investigation, Data curation, Visualization, Writing - original draft. **Jingyang Liu:** Resources, Formal analysis, Data curation, Visualization, Writing - original draft. **Weiping Wang:** Resources, Methodology, Validation. **Jiangfeng Li:** Software, Formal analysis, Data curation. **Guangming Liu:** Resources, Validation. **Ruiqi Qiu:** Resources, Writing - review & editing. **Mingzhu Yang:** Resources, Investigation. **Meng Liu:** Validation, Visualization. **Lin Yang:** Investigation, Visualization. **Xiaofeng Du:** Resources. **Bo Lei:** Conceptualization, Resources, Writing - review & editing, Supervision, Project administration, Funding acquisition. All authors have read and approved the final manuscript.

## Supplementary material

Supplementary Figure S1 **Samples for proteome analysis A.** Immunofluorescence staining of the hRPE cells with antibody against RPE65. RPE-specific antibody, anti-human RPE65 confirms that the cells we collected from donor's eyes were RPE-derived cell lines. RPE65 is located in the cytoplasm around the DAPI-positive nucleus. Both hRPE and ARPE-19 cells displayed similar morphology and RPE65 staining results. **B**. The detailed sample assignments for TMT10 labeling. Eighteen intracellular samples were randomly labeled with TMT-10plex labeling reagents, and their mixture was labeled by TMT131 as an internal standard in each batch of TMT-10plex. Secreted samples were labeled in the same way as intra-cellular sample assignments. **C**. and **D**. Boxplots of the samples in MS quantification. Boxplots indicate 75% interquartile range, median, and outliers of the intra-cellular (**C**) and secreted (**D**) samples after a between-sample normalization by the median of intensities.

Supplementary Figure S2 **Quality control and basic features of RPE proteome** Quality controls of the protein quantification, including the number of intracellular (**A**) and secreted (**C**) proteins, and CV of the intra-cellular (**B**) and secreted (**D**) proteins in the Y, M, and O groups. The number of quantified proteins and the median CV values for the Y, M, and O groups were comparable. **E**. Subcellular location analysis of the intracellular proteins according to GO database. Numbers of the quantified proteins are indicated in round brackets below the subcellular type. **F**. Venn diagram depicting the overlap between secreted proteins (or vesicles) and the quantified proteins in our results. Secreted proteins or vesicles were predicated based on the mRNA level according to the human protein atlas database-the cell atlas. CV, coefficient of variation; GO, gene oncology.

Supplementary Figure S3 **Function analysis of RNF123, RNF149, and protein ubiquitination** Relative grey date of protein ubiquitination, RNF123, and RNF149 levels in ARPE-19 (**A**) and HEK293T (**B**) cells, stimulated with gradient concentrations of D-gal for 4 h. Relative grey date of protein ubiquitination, RNF123, and RNF149 levels in ARPE-19 (**C**) and HEK293T (**D**) cells, stimulated with gradient concentrations of H_2_O_2_ for 30 min. RNF123, RNF149, and the ubiquitinated proteins were up-regulated in ARPE-19 cells with increasing concentrations of H_2_O_2_, and this phenomenon is partially reversible by lutein. **E**. Cell viability of hRPE cells with different ages after H_2_O_2_ stimulation. hRPE cells of O donors were more vulnerable to H_2_O_2_ stimulation. **F**. WB analysis of the O and Y hRPE cells confirms the H_2_O_2_ stimulation is related to the up-regulation of protein ubiquitylation. 67-year-old donor for the old, and 13-year-old donor for the young. Relative grey date of protein ubiquitination, RNF123 or RNF149 levels in ARPE-19 and HEK293T cells, with overexpressing RNF149 (**G**) or RNF123 (**H**). **I**. ROS production in ARPE-19 cells treated with H_2_O_2_. H_2_O_2_-induced ROS generation was significantly inhibited by RNF123 or RNF149 overexpression. ROS, reactive oxygen species; ns, no significance; *, *P* < 0.05; **, *P* < 0.01; ***, *P* < 0.005.

Supplementary Figure S4 **Information on age-associated secretory phenotype A**. Significant discrete clusters to illustrate the relative expression changes of the secreted proteomics. Clusters were clustered by mfuzz. **B**. WGCNA cluster dendrogram generated by an unsupervised hierarchical analysis of all quantified secreted proteins. The analysis was based on topological overlap followed by branch cutting and revealed 15 modules coded in different colors. **C**. GO (biological process) network depiction of protein co-expression modules. The edges (lines) represent connections between the nodes, and nodes indicate proteins. Blue represents M7 (positive correlations), green represents M9 (negative correlations), and red represents M11 (positive correlations). WGCNA, weighted gene co-expression network analysis.

Supplementary Table S1 **hRPE cell donor information**

Supplementary Table S2 **Quantitative proteomic profiling of the hRPE intra-cellular and secreted proteins**

Supplementary Table S3 **Summary of the cluster and GSEA analysis of the intracellular proteins**

Supplementary Table S4 **Summary of the age-associated intra-cellular proteins and their functional analysis**

Supplementary Table S5 **Summary of the functional analysis of the secreted proteins**

Supplementary Table S6 **Summary of the specifically changed secreted proteins and their functional analysis**
