# Supplementary figures and images for: Identification of Age-associated Proteins and Functional Alterations in Human Retinal Pigment Epithelium
Source: Genomics Proteomics Bioinformatics. 2022 Jun 23;20(4):633–47. doi: 10.1016/j.gpb.2022.06.001 (PMC9880895; doi:10.1016/j.gpb.2022.06.001)

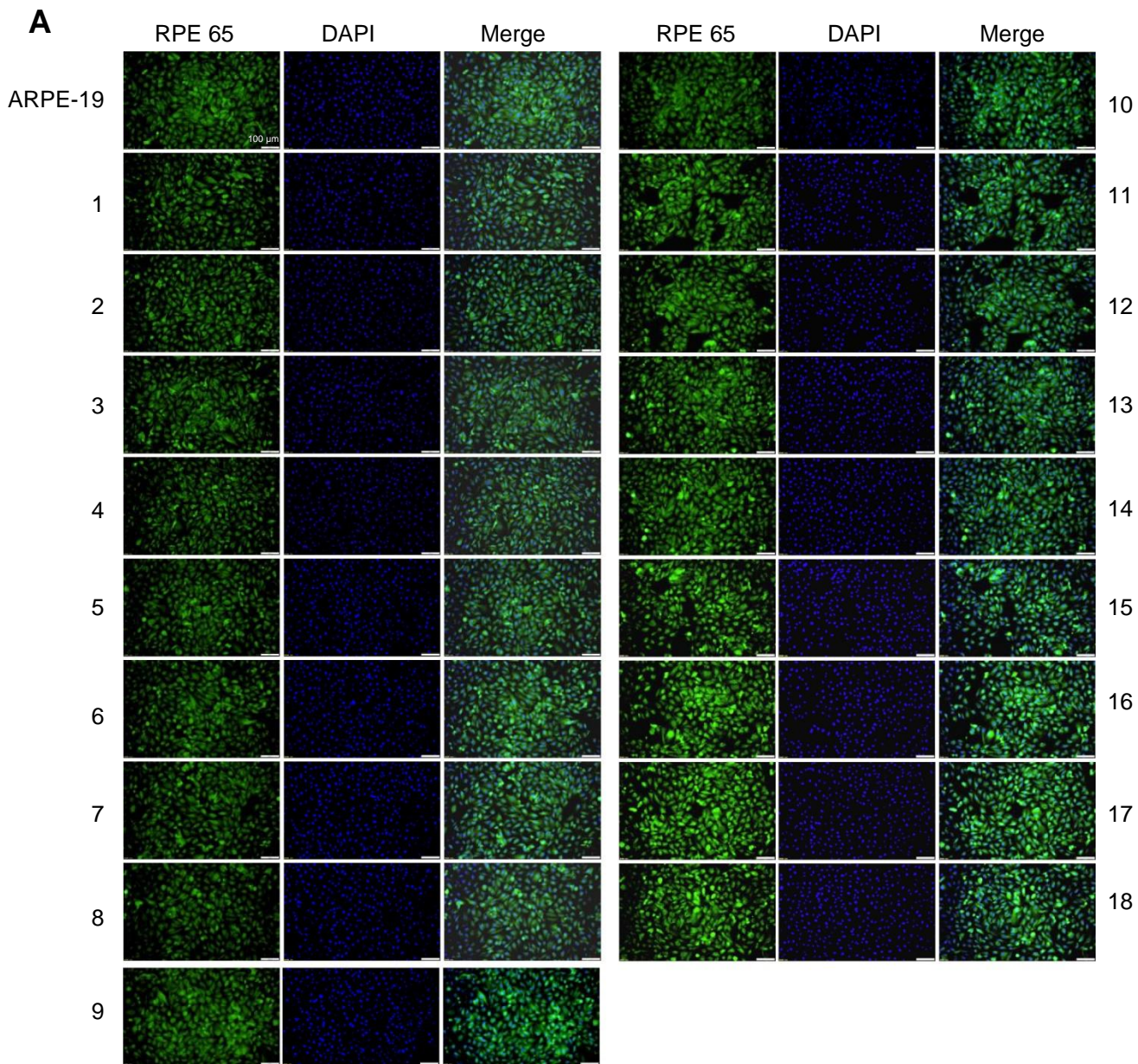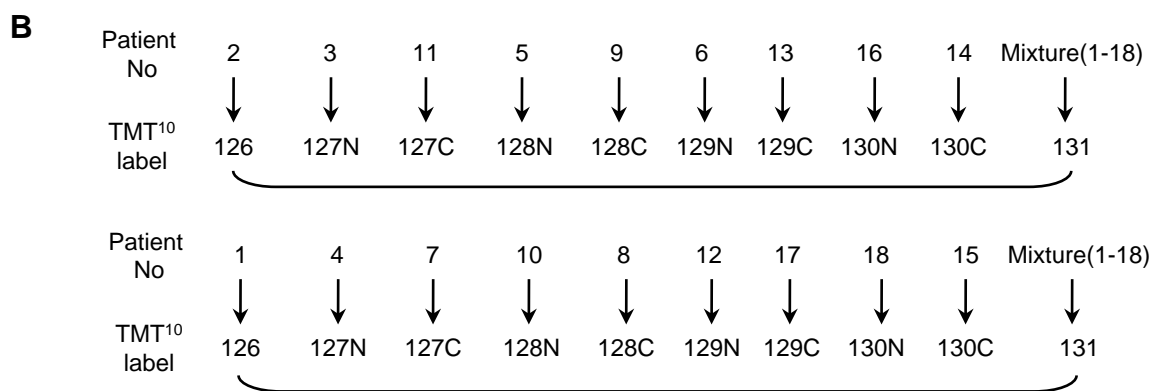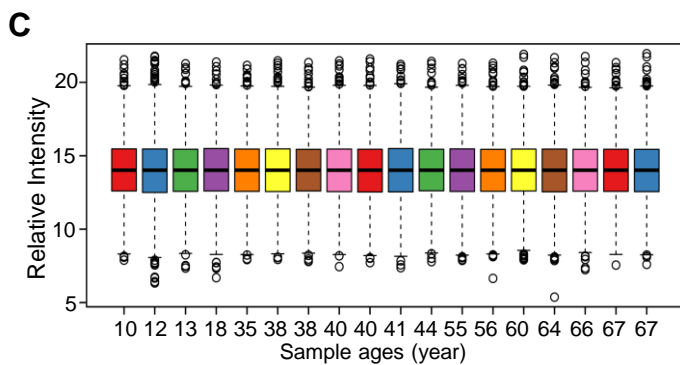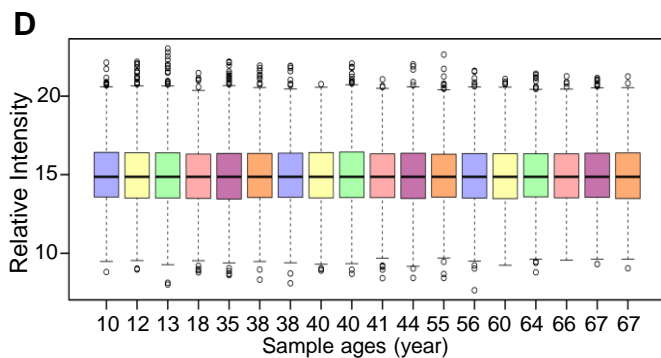

Supplement: Supplementary Figure S1 — Samples for proteome analysis A. Immunofluorescence staining of the hRPE cells with antibody against RPE65. RPE-specific antibody, anti-human RPE65 confirms that the cells we collected from donor's eyes were RPE-derived cell lines. RPE65 is located in the cytoplasm around the DAPI-positive nucleus. Both hRPE and ARPE-19 cells displayed similar morphology and RPE65 staining results. B. The detailed sample assignments for TMT10 labeling. Eighteen intracellular samples were randomly labeled with TMT-10plex labeling reagents, and their mixture was labeled by TMT131 as an internal standard in each batch of TMT-10plex. Secreted samples were labeled in the same way as intra-cellular sample assignments. C. and D. Boxplots of the samples in MS quantification. Boxplots indicate 75% interquartile range, median, and outliers of the intra-cellular (C) and secreted (D) samples after a between-sample normalization by the median of intensities. [file mmc1.pdf]

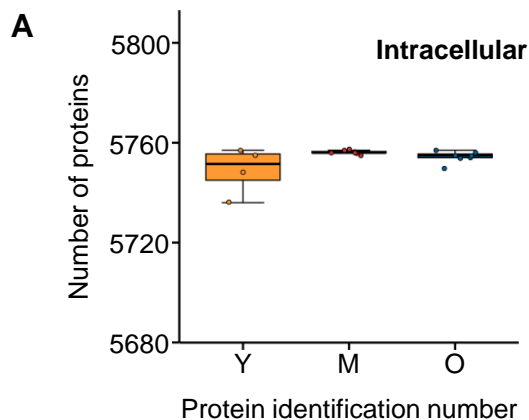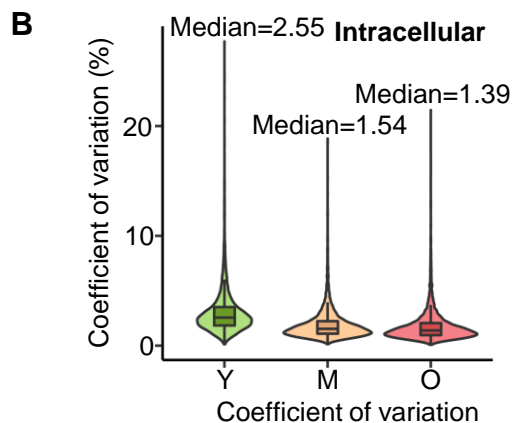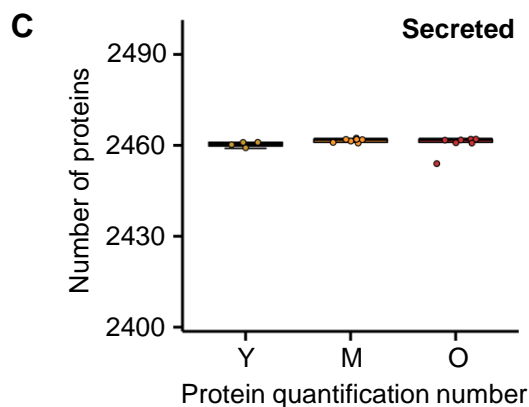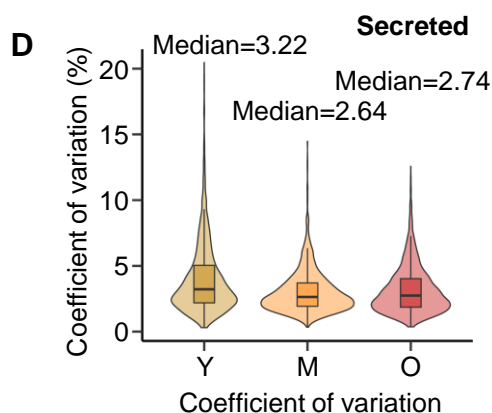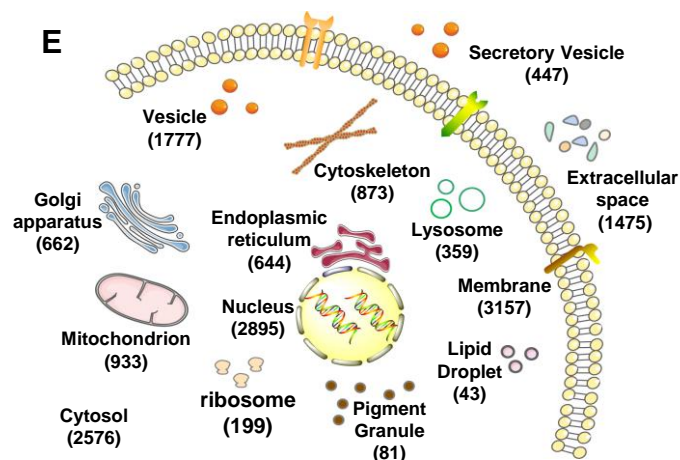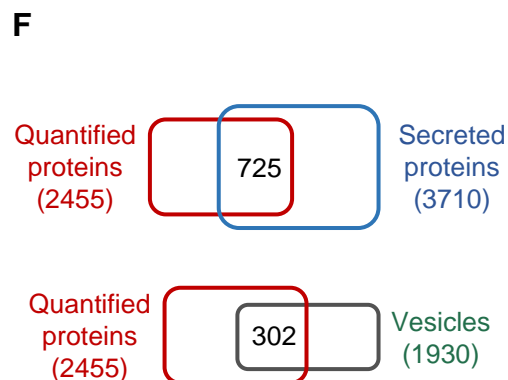

Supplement: Supplementary Figure S2 — Quality control and basic features of RPE proteome Quality controls of the protein quantification, including the number of intracellular (A) and secreted (C) proteins, and CV of the intra-cellular (B) and secreted (D) proteins in the Y, M, and O groups. The number of quantified proteins and the median CV values for the Y, M, and O groups were comparable. E. Subcellular location analysis of the intracellular proteins according to GO database. Numbers of the quantified proteins are indicated in round brackets below the subcellular type. F. Venn diagram depicting the overlap between secreted proteins (or vesicles) and the quantified proteins in our results. Secreted proteins or vesicles were predicated based on the mRNA level according to the human protein atlas database-the cell atlas. CV, coefficient of variation; GO, gene oncology. [file mmc2.pdf]

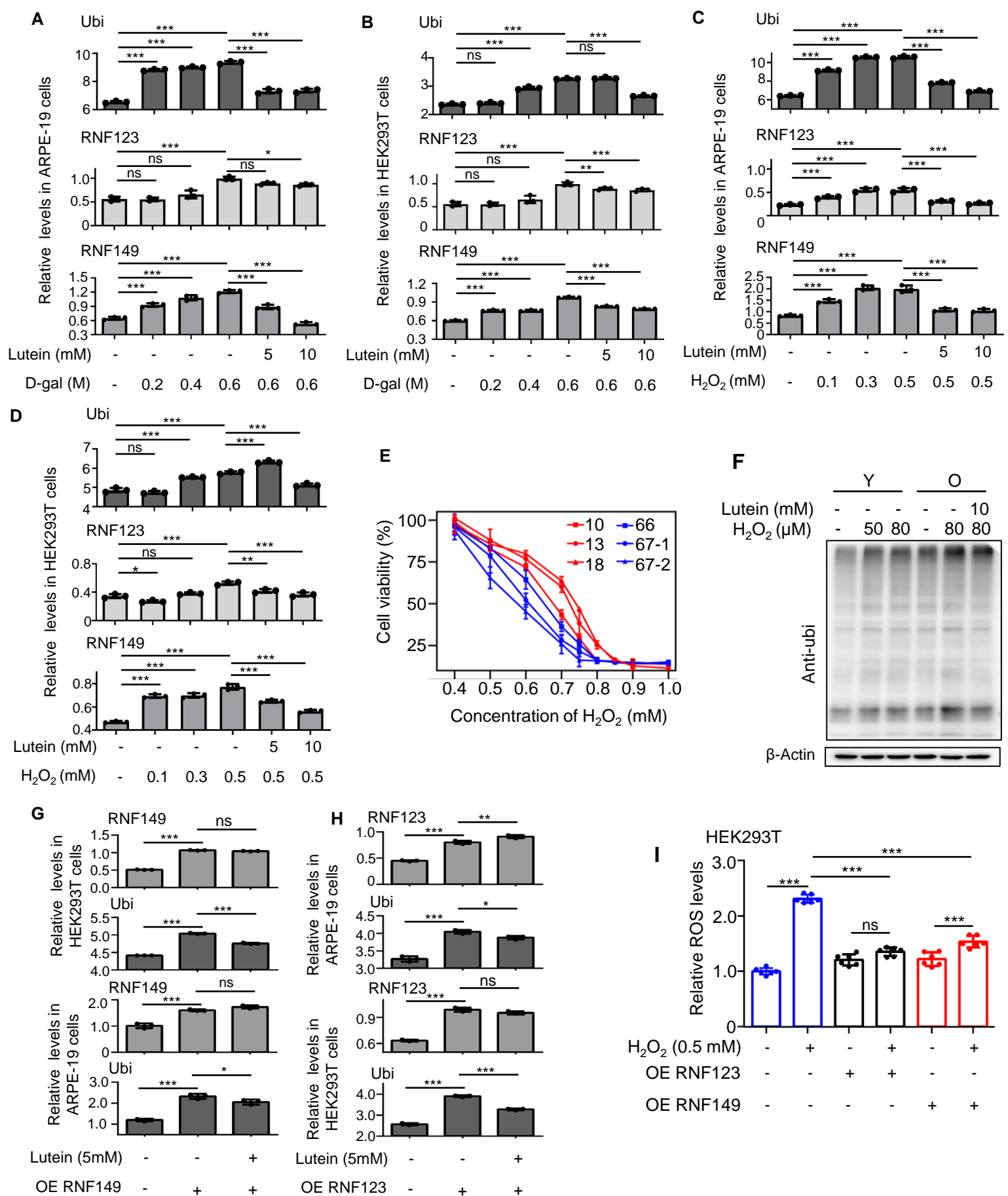

Supplement: Supplementary Figure S3 — Function analysis of RNF123, RNF149, and protein ubiquitination Relative grey date of protein ubiquitination, RNF123, and RNF149 levels in ARPE-19 (A) and HEK293T (B) cells, stimulated with gradient concentrations of D-gal for 4 h. Relative grey date of protein ubiquitination, RNF123, and RNF149 levels in ARPE-19 (C) and HEK293T (D) cells, stimulated with gradient concentrations of H2O2 for 30 min. RNF123, RNF149, and the ubiquitinated proteins were up-regulated in ARPE-19 cells with increasing concentrations of H2O2, and this phenomenon is partially reversible by lutein. E. Cell viability of hRPE cells with different ages after H2O2 stimulation. hRPE cells of O donors were more vulnerable to H2O2 stimulation. F. WB analysis of the O and Y hRPE cells confirms the H2O2 stimulation is related to the up-regulation of protein ubiquitylation. 67-year-old donor for the old, and 13-year-old donor for the young. Relative grey date of protein ubiquitination, RNF123 or RNF149 levels in ARPE-19 and HEK293T cells, with overexpressing RNF149 (G) or RNF123 (H). I. ROS production in ARPE-19 cells treated with H2O2. H2O2-induced ROS generation was significantly inhibited by RNF123 or RNF149 overexpression. ROS, reactive oxygen species; ns, no significance; *, P < 0.05; **, P < 0.01; ***, P < 0.005. [file mmc3.pdf]

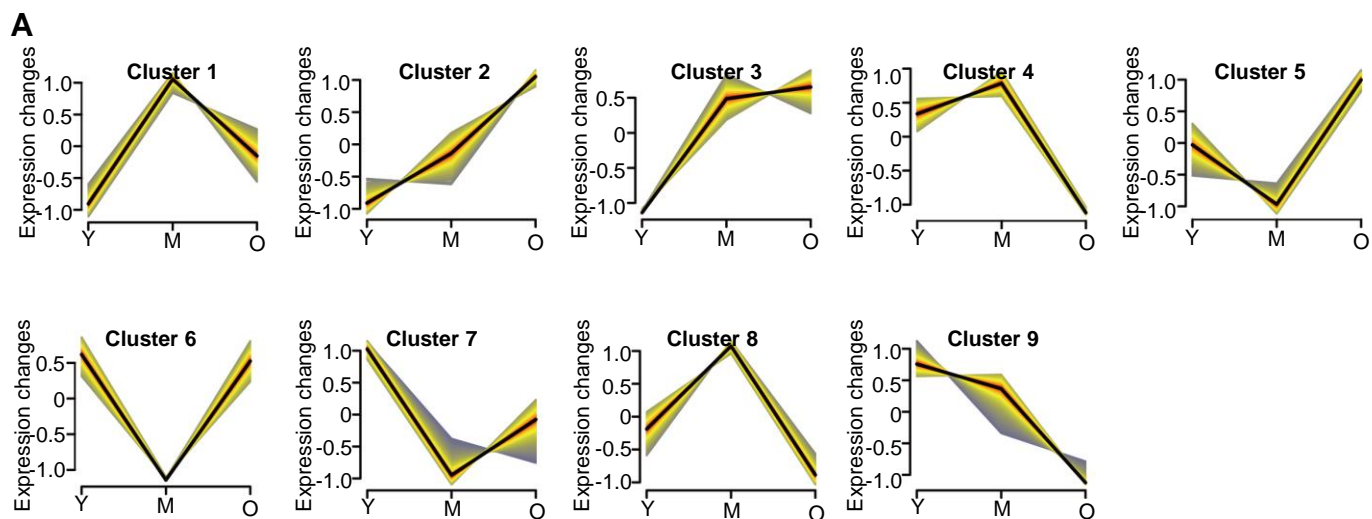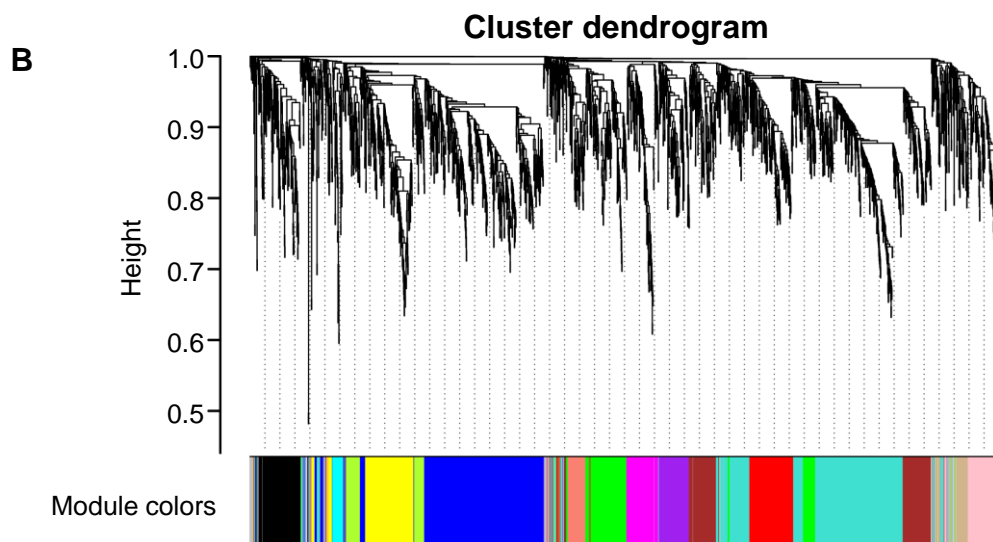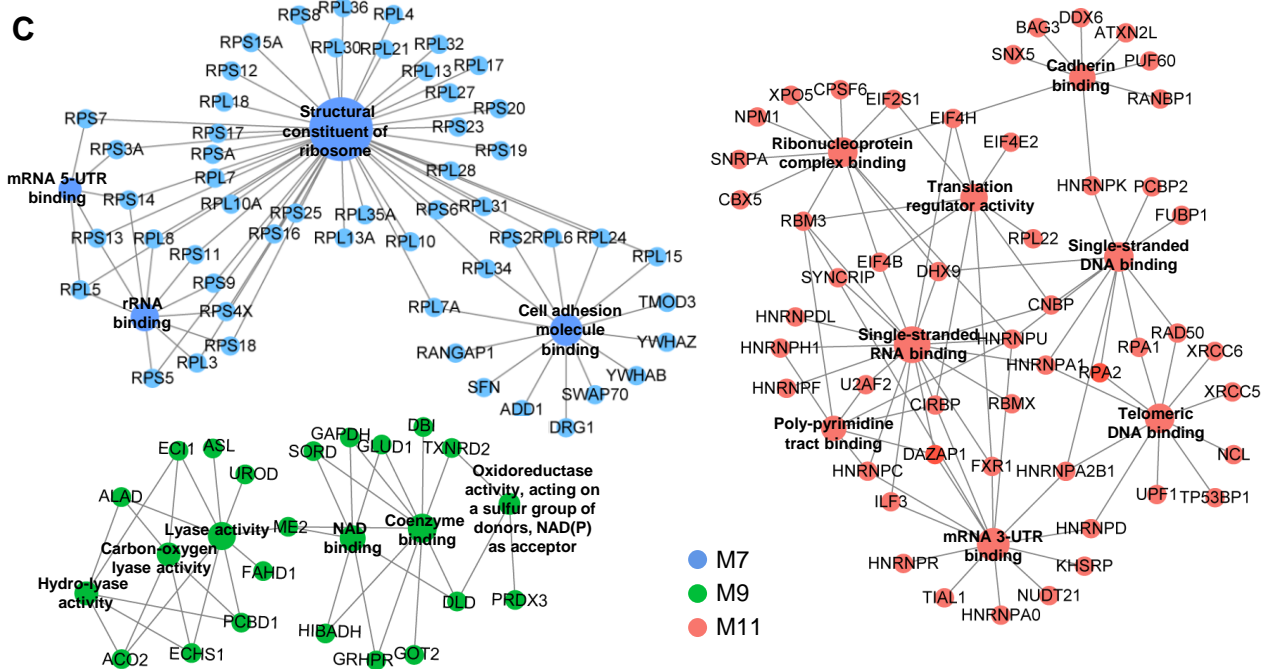

Supplement: Supplementary Figure S4 — Information on age-associated secretory phenotype A. Significant discrete clusters to illustrate the relative expression changes of the secreted proteomics. Clusters were clustered by mfuzz. B. WGCNA cluster dendrogram generated by an unsupervised hierarchical analysis of all quantified secreted proteins. The analysis was based on topological overlap followed by branch cutting and revealed 15 modules coded in different colors. C. GO (biological process) network depiction of protein co-expression modules. The edges (lines) represent connections between the nodes, and nodes indicate proteins. Blue represents M7 (positive correlations), green represents M9 (negative correlations), and red represents M11 (positive correlations). WGCNA, weighted gene co-expression network analysis. [file mmc4.pdf]
